# Use of genomic DNA control features and predicted operon structure in microarray data analysis: ArrayLeaRNA – a Bayesian approach

**DOI:** 10.1186/1471-2105-8-455

**Published:** 2007-11-19

**Authors:** Carmen Pin, Mark Reuter

**Affiliations:** 1 Institute of Food Research, Norwich, NR4 7UA, UK

## Abstract

**Background:**

Microarrays are widely used for the study of gene expression; however deciding on whether observed differences in expression are significant remains a challenge.

**Results:**

A computing tool (ArrayLeaRNA) has been developed for gene expression analysis. It implements a Bayesian approach which is based on the Gumbel distribution and uses printed genomic DNA control features for normalization and for estimation of the parameters of the Bayesian model and prior knowledge from predicted operon structure. The method is compared with two other approaches: the classical LOWESS normalization followed by a two fold cut-off criterion and the OpWise method (Price, et al. 2006. BMC Bioinformatics. 7, 19), a published Bayesian approach also using predicted operon structure. The three methods were compared on experimental datasets with prior knowledge of gene expression. With ArrayLeaRNA, data normalization is carried out according to the genomic features which reflect the results of equally transcribed genes; also the statistical significance of the difference in expression is based on the variability of the equally transcribed genes. The operon information helps the classification of genes with low confidence measurements.

ArrayLeaRNA is implemented in Visual Basic and freely available as an Excel add-in at

**Conclusion:**

We have introduced a novel Bayesian model and demonstrated that it is a robust method for analysing microarray expression profiles. ArrayLeaRNA showed a considerable improvement in data normalization, in the estimation of the experimental variability intrinsic to each hybridization and in the establishment of a clear boundary between non-changing and differentially expressed genes. The method is applicable to data derived from hybridizations of labelled cDNA samples as well as from hybridizations of labelled cDNA with genomic DNA and can be used for the analysis of datasets where differentially regulated genes predominate.

## Background

DNA microarrays are well established means of monitoring genome-wide patterns of gene expression [1]. The first level of analysis requires determining whether observed differences in expression are significant. Data analysis techniques are actively being developed for this purpose including classical ANOVA methods [2,3] and Bayesian approaches based on both Gaussian [4-9] and non-Gaussian [10,11] models. Several problems arise with classical statistical inference due to the lack of replications and the large amount of genes in cases of multiple testing [6]. None of the existing methods estimate the variability of the measurements from equally expressed genes. Our goal is to introduce a new Bayesian approach for transcriptome analysis, ArrayLeaRNA, based on the intrinsic variability of the equally expressed genes estimated from genomic DNA features printed on the microarray slide and on the predicted transcriptional organisation of operons. The model underlying ArrayLeaRNA assumes that the log ratios have a Gumbel distribution; this gives an asymmetric posterior distribution with a very steep tail, which is more discerning than that obtained from the Gaussian model.

To test ArrayLeaRNA, we compared it with two other analysis approaches: a constant two-fold cut-off value, i.e. two-fold changes between intensities, and OpWise [8]. The reasons for the choice of these two methods are that the two-fold cut-off is a common practice for data analysis with some commercial analysis packages and OpWise is a Bayesian approach based on a Gaussian model, as used in other published approaches [4-9]. OpWise also incorporates predicted operon structure to inform on systematic microarray errors and to decide whether the expression is significantly different. The performance of these analysis approaches is illustrated using three experimental hybridization datasets with prior knowledge of expected transcription patterns.

We introduce the use of genomic DNA features printed as serial dilutions on the microarray slide and demonstrate that the measurements from these features are equivalent to the measurements of genes that are equally expressed under different experimental conditions. The normalization approach presented in this paper, based on these genomic control features, was compared with the so-called LOWESS normalization [12] based on the LOWESS non parametric regression [13] applied to describe the relationship between the difference (M) and the average (A) of the logarithm of the intensities. We use the genomic controls not only in data normalization, but also in data analysis for the estimation of the parameters of the Bayesian model. Also the Bayesian model includes the information on the transcription of the predicted operon to help the correct assignment of genes with low confidence measurements. ArrayLeaRNA is implemented in a new user-friendly software tool freely available [14].

### Description of the analysis

#### Hybridization datasets analyzed in this study

(Datasets are available at [14])

Dataset I is the result of a microarray hybridization of cDNA obtained from *C. jejuni *strains 11168 and 81116 and labelled with Cy3 and Cy5, respectively. There are 6 replicates for each ORF and ca. 8–10 replicates for each genomic DNA control feature (100, 250, 500, 1000, 3000 and 5000 ng) from each strain.

Dataset II was obtained from a hybridization of cDNA made from two replicated cultures of *S. pneumoniae *TIGR 4. The samples of cDNA were differentially labelled with Cy3 and Cy5. The dataset contains ca. 4 replicated measurements from each ORF and ca. 15 replicated measurements of each genomic DNA feature (10, 50, 100, 500 and 1000 ng).

Dataset III was obtained from two independent hybridizations. Each hybridization was carried out by mixing genomic DNA with cDNA both obtained from *E. coli*. The mixture was hybridized to the microarray slide. Dataset III was constructed by combining the fluorescence intensities measured from the cDNA sample in each hybridization. The dataset consisted of one measurement of each ORF and ca. 15 replicated measurements of each genomic DNA feature (25, 75, 250, 750 and 2250 ng).

Dataset IV was generated from Dataset I. The log ratios of differentially expressed genes were made positive so that 178 genes were up-regulated in sample 1 and none in sample 2. Then, a set of 178 equally transcribed genes were randomly selected. This gives an asymmetric gene expression dataset with 356 genes from which 178 genes up-regulated in sample 1 and 178 genes equally transcribed, and therefore the mean ratio of the whole dataset is very different from the mean ratio of the equally transcribed genes.

### Standardization of the hybridization datasets

Data standardization was based on the genomic DNA features printed on the microarray slides at different concentrations. We reasoned that equal amounts of each differentially labelled cDNA sample will hybridize to the genomic control features and can therefore be used as a reference to standardize the whole hybridization dataset. In order to do this, the relationship between the intensities measured on the genomic features is described as:

log_2 _*I*_1 _= *α*_*g *_+ *β*_*g *_log_2_*I*_2_

where *I*_1 _and *I*_2 _are the fluorescence intensities observed from each sample differentially labelled and hybridized on one slide in the case of cDNA *vs *cDNA hybridizations. For cDNA *vs *genomic DNA hybridizations, *I*_1 _and *I*_2 _are the intensities corresponding to the cDNA samples measured in two different slides.

The standardization of the intensities was based on both parameters *α*_*g *_and *β*_*g *_specifically estimated for each microarray hybridization. Both the intensities measured from ORFs and genomic features were corrected as follows:

log_2 _*I*_1_' = *β*_*g *_log_2 _*I*_1 _+ *α*_*g*_

log_2 _*I*_2_' = log_2 _*I*_2_

The value of 2^*αg*^, the constant of proportionality between intensities, should in theory be 1, i.e. *α*_*g *_= 0, because equal amounts of differentially labelled cDNA should hybridize to the genomic features. A value of *β*_*g *_different from 1 expresses lack of linearity between the intensities. To avoid unnecessary non-linear data transformation, *β*_*g *_took a value different from 1 only if an F test rejected the null hypothesis *β*_*g *_= 1.

After standardization, the average of the logarithm of the ratios between the intensities measured from the genomic features was equal to zero. Accordingly, the logarithm of the ratios measured from ORFs with equal amounts of transcripts of both samples hybridized is also expected to be centred on zero.

### Bayesian inference test

We denote the logarithm of the ratio between intensities as *R *= log_2 _(*I*_1_) - log_2_(*I*_2_), where *I*_1 _and *I*_2 _are the fluorescence intensities measured for two differentially labelled samples of cDNA. Samples 1 and 2 are either hybridized on one slide in the case of cDNA-cDNA hybridizations or on two slides in the case of cDNA-DNA hybridizations.

We assume that *R *has a Gumbel distribution with density function

gR(r)=1beA−rbe−eA−rb−∞<r<∞
 MathType@MTEF@5@5@+=feaafiart1ev1aaatCvAUfKttLearuWrP9MDH5MBPbIqV92AaeXatLxBI9gBaebbnrfifHhDYfgasaacPC6xNi=xI8qiVKYPFjYdHaVhbbf9v8qqaqFr0xc9vqFj0dXdbba91qpepeI8k8fiI+fsY=rqGqVepae9pg0db9vqaiVgFr0xfr=xfr=xc9adbaqaaeGacaGaaiaabeqaaeqabiWaaaGcbaqbaeqabeGaaaqaaiabdEgaNnaaBaaaleaacqWGsbGuaeqaaOGaeiikaGIaemOCaiNaeiykaKIaeyypa0tcfa4aaSaaaeaacqaIXaqmaeaacqWGIbGyaaGccqWGLbqzdaahaaWcbeqcfayaamaalaaabaGaemyqaeKaeyOeI0IaemOCaihabaGaemOyaigaaaaakiabdwgaLnaaCaaaleqabaGaeyOeI0Iaemyzau2aaWbaaWqabKqbagaadaWcaaqaaiabdgeabjabgkHiTiabdkhaYbqaaiabdkgaIbaaaaaaaaGcbaaccaGae8NeI0IaeyOhIuQaeyipaWJaemOCaiNaeyipaWJaeyOhIukaaaaa@4D5B@

where *A *and *b *are the location and scale parameters, respectively. After an experiment, there is uncertainty concerning the location parameter, *A*, which has a prior distribution *ξ*_*A prior*_. The parameter *b *is invariant and known using the genomic control features. When *b *is known a conjugate family of distributions for the parameter *A *exists (see the Appendix).

We wish to know whether a gene is differentially expressed. For genes that show similar expression in both samples, the ratios are fairly close to 1 and the value of the location parameter, *A*, is centred on a value *a*_0_. To avoid an arbitrary asymmetry in the results, the hypothesis test is made by using only one tail of the posterior distribution of *A*. Additionally, the information on the transcription pattern of the operon to which the gene belongs is to be used in the analysis. For these reasons we chose an informative prior for *A *given by:

ξA prior(a)=|p1Cf(a;α,β)if −∞<a<a0p0Cf(a;α,β)if a0≤a<∞
 MathType@MTEF@5@5@+=feaafiart1ev1aaatCvAUfKttLearuWrP9MDH5MBPbIqV92AaeXatLxBI9gBaebbnrfifHhDYfgasaacPC6xNi=xI8qiVKYPFjYdHaVhbbf9v8qqaqFr0xc9vqFj0dXdbba91qpepeI8k8fiI+fsY=rqGqVepae9pg0db9vqaiVgFr0xfr=xfr=xc9adbaqaaeGacaGaaiaabeqaaeqabiWaaaGcbaacciGae8NVdG3aaSbaaSqaaiabdgeabjabbccaGiabdchaWjabdkhaYjabdMgaPjabd+gaVjabdkhaYbqabaGccqGGOaakcqWGHbqycqGGPaqkcqGH9aqpdaabbaqaauaabaqaciaaaeaajuaGdaWcaaqaaiabdchaWnaaBaaabaGaeGymaedabeaaaeaacqWGdbWqaaGccqWGMbGzcqGGOaakcqWGHbqycqGG7aWocqWFXoqycqGGSaalcqWFYoGycqGGPaqkaeaacqqGPbqAcqqGMbGzcqqGGaaiiiaacqGFsislcqGHEisPcqGH8aapcqWGHbqycqGH8aapcqWGHbqydaWgaaWcbaGaeGimaadabeaaaOqaaKqbaoaalaaabaGaemiCaa3aaSbaaeaacqaIWaamaeqaaaqaaiabdoeadbaakiabdAgaMjabcIcaOiabdggaHjabcUda7iab=f7aHjabcYcaSiab=j7aIjabcMcaPaqaaiabbMgaPjabbAgaMjabbccaGiabdggaHnaaBaaaleaacqaIWaamaeqaaOGaeyizImQaemyyaeMaeyipaWJaeyOhIukaaaGaay5bSdaaaa@6EB6@

where f(a;α,β)=1bβαΓ(α)eabαe−βeab
 MathType@MTEF@5@5@+=feaafiart1ev1aqatCvAUfKttLearuWrP9MDH5MBPbIqV92AaeXatLxBI9gBaebbnrfifHhDYfgasaacPC6xNi=xH8viVGI8Gi=hEeeu0xXdbba9frFj0xb9qqpG0dXdb9aspeI8k8fiI+fsY=rqGqVepae9pg0db9vqaiVgFr0xfr=xfr=xc9adbaqaaeGacaGaaiaabeqaaeqabiWaaaGcbaGaemOzayMaeiikaGIaemyyaeMaei4oaSdcciGae8xSdeMaeiilaWIae8NSdiMaeiykaKIaeyypa0tcfa4aaSaaaeaacqaIXaqmaeaacqWGIbGyaaWaaSaaaeaacqWFYoGydaahaaqabeaacqWFXoqyaaaabaGaeu4KdCKaeiikaGIae8xSdeMaeiykaKcaaOGaemyzau2aaWbaaSqabeaajuaGdaWcaaqaaiabdggaHbqaaiabdkgaIbaaliab=f7aHbaakiabdwgaLnaaCaaaleqabaGaeyOeI0Iae8NSdiMaemyzau2aaWbaaWqabKqbagaadaWcaaqaaiabdggaHbqaaiabdkgaIbaaaaaaaaaa@5072@ is described in the Appendix (Eq A3) and *a*_0 _is the centred value of the parameter *A *for those genes equally expressed in both samples; *p*_0 _and *p*_1 _quantify the information of the transcription of the operon and comply with *p*_0 _+ *p*_1 _= 1; C=∫−∞a0p1f(a;α,β)da+∫a0∞p0f(a;α,β)da
 MathType@MTEF@5@5@+=feaafiart1ev1aqatCvAUfKttLearuWrP9MDH5MBPbIqV92AaeXatLxBI9gBaebbnrfifHhDYfgasaacPC6xNi=xH8viVGI8Gi=hEeeu0xXdbba9frFj0xb9qqpG0dXdb9aspeI8k8fiI+fsY=rqGqVepae9pg0db9vqaiVgFr0xfr=xfr=xc9adbaqaaeGacaGaaiaabeqaaeqabiWaaaGcbaGaem4qamKaeyypa0Zaa8qmaeaacqWGWbaCdaWgaaWcbaGaeGymaedabeaakiabdAgaMjabcIcaOiabdggaHjabcUda7GGaciab=f7aHjabcYcaSiab=j7aIjabcMcaPiabdsgaKjabdggaHjabgUcaRaWcbaGaeyOeI0IaeyOhIukabaGaemyyae2aaSbaaWqaaiabicdaWaqabaaaniabgUIiYdGcdaWdXaqaaiabdchaWnaaBaaaleaacqaIWaamaeqaaOGaemOzayMaeiikaGIaemyyaeMaei4oaSJae8xSdeMaeiilaWIae8NSdiMaeiykaKIaemizaqMaemyyaegaleaacqWGHbqydaWgaaadbaGaeGimaadabeaaaSqaaiabg6HiLcqdcqGHRiI8aaaa@58F9@ is a normalizing constant such that ∫−∞∞ξ(a)priorda=1
 MathType@MTEF@5@5@+=feaafiart1ev1aqatCvAUfKttLearuWrP9MDH5MBPbIqV92AaeXatLxBI9gBaebbnrfifHhDYfgasaacPC6xNi=xH8viVGI8Gi=hEeeu0xXdbba9frFj0xb9qqpG0dXdb9aspeI8k8fiI+fsY=rqGqVepae9pg0db9vqaiVgFr0xfr=xfr=xc9adbaqaaeGacaGaaiaabeqaaeqabiWaaaGcbaWaa8qmaeaaiiGacqWF+oaEdaWgbaWcbaGaemiCaaNaemOCaiNaemyAaKMaem4Ba8MaemOCaihabeaakiabcIcaOiabdggaHjabcMcaPiabdsgaKjabdggaHjabg2da9iabigdaXaWcbaGaeyOeI0IaeyOhIukabaGaeyOhIukaniabgUIiYdaaaa@4261@.

Bayes theorem gives a posterior distribution for *A *as:

$$\xi_{A\;post}\left(a|r_{1}..r_{n}\right)=|\begin{array}{ll} \frac{p_{1}g\left(r_{1}..r_{n}|a\right)f(a)}{p_{1}\int_{-\infty }^{a_{0}}g\left(r_{1}..r_{n}|a\right)f(a)da+p_{0}\int_{a_{0}}^{\infty }g\left(r_{1}..r_{n}|a\right)f(a)da} & \text{if }-\infty <a<a_{0}\\ \frac{p_{0}g\left(r_{1}..r_{n}|a\right)f(a)}{p_{1}\int_{-\infty }^{a_{0}}g\left(r_{1}..r_{n}|a\right)f(a)da+p_{0}\int_{a_{0}}^{\infty }g\left(r_{1}..r_{n}|a\right)f(a)da} & \text{if }a_{0}\leq a<\infty \end{array}$$

where g(r1..rn|a)=1bnenabe−∑i=1nribe−k˜eab
 MathType@MTEF@5@5@+=feaafiart1ev1aqatCvAUfKttLearuWrP9MDH5MBPbIqV92AaeXatLxBI9gBaebbnrfifHhDYfgasaacPC6xNi=xH8viVGI8Gi=hEeeu0xXdbba9frFj0xb9qqpG0dXdb9aspeI8k8fiI+fsY=rqGqVepae9pg0db9vqaiVgFr0xfr=xfr=xc9adbaqaaeGacaGaaiaabeqaaeqabiWaaaGcbaGaem4zaCMaeiikaGIaemOCai3aaSbaaSqaaiabigdaXaqabaGccqGGUaGlcqGGUaGlcqWGYbGCdaWgaaWcbaGaemOBa4gabeaakmaaeeaabaGaemyyaegacaGLhWoacqGGPaqkcqGH9aqpjuaGdaWcaaqaaiabigdaXaqaaiabdkgaInaaCaaabeqaaiabd6gaUbaaaaGccqWGLbqzdaahaaWcbeqcfayaamaalaaabaGaemOBa4MaemyyaegabaGaemOyaigaaaaakiabdwgaLnaaCaaaleqabaGaeyOeI0YaaabCaKqbagaadaWcaaqaaiabdkhaYnaaBaaabaGaemyAaKgabeaaaeaacqWGIbGyaaaameaacqWGPbqAcqGH9aqpcqaIXaqmaeaacqWGUbGBa4GaeyyeIuoaaaGccqWGLbqzdaahaaWcbeqaaiabgkHiTmaaGaaabaGaem4AaSgacaGLdmaacqWGLbqzdaahaaadbeqcfayaamaalaaabaGaemyyaegabaGaemOyaigaaaaaaaaaaa@5C40@ is the density function of the sample of measurements conditioned by *A *= *a *as described in the Appendix (Eq A2); *n *is the number of independent measurements for the gene in study and k˜
 MathType@MTEF@5@5@+=feaafiart1ev1aqatCvAUfKttLearuWrP9MDH5MBPbIqV92AaeXatLxBI9gBaebbnrfifHhDYfgasaacPC6xNi=xH8viVGI8Gi=hEeeu0xXdbba9frFj0xb9qqpG0dXdb9aspeI8k8fiI+fsY=rqGqVepae9pg0db9vqaiVgFr0xfr=xfr=xc9adbaqaaeGacaGaaiaabeqaaeqabiWaaaGcbaWaaacaaeaacqWGRbWAaiaawoWaaaaa@2DF5@ is a sample statistic: k˜(r1..rn)=n⋅e| r¯/b|
 MathType@MTEF@5@5@+=feaafiart1ev1aqatCvAUfKttLearuWrP9MDH5MBPbIqV92AaeXatLxBI9gBaebbnrfifHhDYfgasaacPC6xNi=xH8viVGI8Gi=hEeeu0xXdbba9frFj0xb9qqpG0dXdb9aspeI8k8fiI+fsY=rqGqVepae9pg0db9vqaiVgFr0xfr=xfr=xc9adbaqaaeGacaGaaiaabeqaaeqabiWaaaGcbaWaaacaaeaacqWGRbWAaiaawoWaaiabcIcaOiabdkhaYnaaBaaaleaacqaIXaqmaeqaaOGaeiOla4IaeiOla4IaemOCai3aaSbaaSqaaiabd6gaUbqabaGccqGGPaqkcqGH9aqpcqWGUbGBcqGHflY1cqWGLbqzdaahaaWcbeqaamaaemaabaWaaSGbaeaacqqGGaaicuWGYbGCgaqeaaqaaiabdkgaIbaaaiaawEa7caGLiWoaaaaaaa@4410@ where r¯
 MathType@MTEF@5@5@+=feaafiart1ev1aqatCvAUfKttLearuWrP9MDH5MBPbIqV92AaeXatLxBI9gBaebbnrfifHhDYfgasaacPC6xNi=xH8viVGI8Gi=hEeeu0xXdbba9frFj0xb9qqpG0dXdb9aspeI8k8fiI+fsY=rqGqVepae9pg0db9vqaiVgFr0xfr=xfr=xc9adbaqaaeGacaGaaiaabeqaaeqabiWaaaGcbaGafmOCaiNbaebaaaa@2D59@ is the mean value of the ratios measured for the gene in study. k˜
 MathType@MTEF@5@5@+=feaafiart1ev1aqatCvAUfKttLearuWrP9MDH5MBPbIqV92AaeXatLxBI9gBaebbnrfifHhDYfgasaacPC6xNi=xH8viVGI8Gi=hEeeu0xXdbba9frFj0xb9qqpG0dXdb9aspeI8k8fiI+fsY=rqGqVepae9pg0db9vqaiVgFr0xfr=xfr=xc9adbaqaaeGacaGaaiaabeqaaeqabiWaaaGcbaWaaacaaeaacqWGRbWAaiaawoWaaaaa@2DF5@ is a reformulation of the statistic *k *defined in the Appendix. k˜
 MathType@MTEF@5@5@+=feaafiart1ev1aqatCvAUfKttLearuWrP9MDH5MBPbIqV92AaeXatLxBI9gBaebbnrfifHhDYfgasaacPC6xNi=xH8viVGI8Gi=hEeeu0xXdbba9frFj0xb9qqpG0dXdb9aspeI8k8fiI+fsY=rqGqVepae9pg0db9vqaiVgFr0xfr=xfr=xc9adbaqaaeGacaGaaiaabeqaaeqabiWaaaGcbaWaaacaaeaacqWGRbWAaiaawoWaaaaa@2DF5@ is a function of the absolute value of the average of the ratios so that the sign of the difference between the log intensities do not affect the result. Also, the effect of outlier measurements on the analysis is drastically minimized. k˜
 MathType@MTEF@5@5@+=feaafiart1ev1aqatCvAUfKttLearuWrP9MDH5MBPbIqV92AaeXatLxBI9gBaebbnrfifHhDYfgasaacPC6xNi=xH8viVGI8Gi=hEeeu0xXdbba9frFj0xb9qqpG0dXdb9aspeI8k8fiI+fsY=rqGqVepae9pg0db9vqaiVgFr0xfr=xfr=xc9adbaqaaeGacaGaaiaabeqaaeqabiWaaaGcbaWaaacaaeaacqWGRbWAaiaawoWaaaaa@2DF5@ takes values in the interval [1, 8). The greater the absolute ratio of the intensities, the greater is the probability of *A *to have small values. Thus, the value of *A *is greater in genes equally expressed than in differentially expressed genes. Thus, *P *= *P*(*A *≥ *a*_0_|*r*_1_..*r*_*n*_) is the posterior probability of equal transcription in both samples and it is estimated as

P(A≥a0|r1..rn)=∫a0∞ξA post(a|r1..rn) da=p0p0+p1F(a0;n+α,β+k˜)1−F(a0;n+α,β+k˜)
 MathType@MTEF@5@5@+=feaafiart1ev1aaatCvAUfKttLearuWrP9MDH5MBPbIqV92AaeXatLxBI9gBaebbnrfifHhDYfgasaacPC6xNi=xI8qiVKYPFjYdHaVhbbf9v8qqaqFr0xc9vqFj0dXdbba91qpepeI8k8fiI+fsY=rqGqVepae9pg0db9vqaiVgFr0xfr=xfr=xc9adbaqaaeGacaGaaiaabeqaaeqabiWaaaGcbaGaemiuaaLaeiikaGIaemyqaeKaeyyzImRaemyyae2aaSbaaSqaaiabicdaWaqabaGcdaabbaqaaiabdkhaYnaaBaaaleaacqaIXaqmaeqaaOGaeiOla4IaeiOla4IaemOCai3aaSbaaSqaaiabd6gaUbqabaaakiaawEa7aiabcMcaPiabg2da9maapedabaacciGae8NVdG3aaSbaaSqaaiabdgeabjabbccaGiabdchaWjabd+gaVjabdohaZjabdsha0bqabaGccqGGOaakdaabcaqaaiabdggaHbGaayjcSdGaemOCai3aaSbaaSqaaiabigdaXaqabaGccqGGUaGlcqGGUaGlcqWGYbGCdaWgaaWcbaGaemOBa4gabeaakiabcMcaPiabbccaGaWcbaGaemyyae2aaSbaaWqaaiabicdaWaqabaaaleaacqGHEisPa0Gaey4kIipakiabdsgaKjabdggaHjabg2da9KqbaoaalaaabaGaemiCaa3aaSbaaeaacqaIWaamaeqaaaqaaiabdchaWnaaBaaabaGaeGimaadabeaacqGHRaWkcqWGWbaCdaWgaaqaaiabigdaXaqabaWaaSaaaeaacqWGgbGrdaqadaqaaiabdggaHnaaBaaabaGaeGimaadabeaacqGG7aWocqWGUbGBcqGHRaWkcqWFXoqycqGGSaalcqWFYoGycqGHRaWkdaaiaaqaaiabdUgaRbGaay5adaaacaGLOaGaayzkaaaabaGaeGymaeJaeyOeI0IaemOray0aaeWaaeaacqWGHbqydaWgaaqaaiabicdaWaqabaGaei4oaSJaemOBa4Maey4kaSIae8xSdeMaeiilaWIae8NSdiMaey4kaSYaaacaaeaacqWGRbWAaiaawoWaaaGaayjkaiaawMcaaaaaaaaaaa@87B2@

where F(a0;n+α,β+k˜)=∫−∞a01b(β+k˜)α+nΓ(α+n)eab(α+n)e−(β+k˜)eabda
 MathType@MTEF@5@5@+=feaafiart1ev1aqatCvAUfKttLearuWrP9MDH5MBPbIqV92AaeXatLxBI9gBaebbnrfifHhDYfgasaacPC6xNi=xH8viVGI8Gi=hEeeu0xXdbba9frFj0xb9qqpG0dXdb9aspeI8k8fiI+fsY=rqGqVepae9pg0db9vqaiVgFr0xfr=xfr=xc9adbaqaaeGacaGaaiaabeqaaeqabiWaaaGcbaGaemOray0aaeWaaeaacqWGHbqydaWgaaWcbaGaeGimaadabeaakiabcUda7iabd6gaUjabgUcaRGGaciab=f7aHjabcYcaSiab=j7aIjabgUcaRmaaGaaabaGaem4AaSgacaGLdmaaaiaawIcacaGLPaaacqGH9aqpdaWdXaqaaKqbaoaalaaabaGaeGymaedabaGaemOyaigaamaalaaabaWaaeWaaeaacqWFYoGycqGHRaWkdaaiaaqaaiabdUgaRbGaay5adaaacaGLOaGaayzkaaWaaWbaaeqabaGae8xSdeMaey4kaSIaemOBa4gaaaqaaiabfo5ahjabcIcaOiab=f7aHjabgUcaRiabd6gaUjabcMcaPaaakiabdwgaLnaaCaaaleqabaqcfa4aaSaaaeaacqWGHbqyaeaacqWGIbGyaaWccqGGOaakcqWFXoqycqGHRaWkcqWGUbGBcqGGPaqkaaGccqWGLbqzdaahaaWcbeqaaiabgkHiTmaabmaabaGae8NSdiMaey4kaSYaaacaaeaacqWGRbWAaiaawoWaaaGaayjkaiaawMcaaiabdwgaLnaaCaaameqajuaGbaWaaSaaaeaacqWGHbqyaeaacqWGIbGyaaaaaaaakiabdsgaKjabdggaHbWcbaGaeyOeI0IaeyOhIukabaGaemyyae2aaSbaaWqaaiabicdaWaqabaaaniabgUIiYdaaaa@71A9@ as shown in the Appendix (Eqs A4, A5 and A6). Then, a gene can be declared differentially expressed if its posterior probability is smaller than some predefined cut-off. Throughout this paper, we will use 0.01 as cut-off value

### Parameter estimation

The estimation of the parameters was carried out with the ratios measured from the genomic control features printed on the same slide as the dataset to be analyzed.

The parameter *b *of the Gumbel distribution was estimated by the method of moments as b^=6π2sdg
 MathType@MTEF@5@5@+=feaafiart1ev1aaatCvAUfKttLearuWrP9MDH5MBPbIqV92AaeXatLxBI9gBaebbnrfifHhDYfgasaacPC6xNi=xH8viVGI8Gi=hEeeu0xXdbba9frFj0xb9qqpG0dXdb9aspeI8k8fiI+fsY=rqGqVepae9pg0db9vqaiVgFr0xfr=xfr=xc9adbaqaaeGacaGaaiaabeqaaeqabiWaaaGcbaGafmOyaiMbaKaacqGH9aqpjuaGdaWcaaqaamaakaaabaGaeGOnaydabeaaaeaaiiGacqWFapaCaaGccqaIYaGmcqWGZbWCcqWGKbazdaWgaaWcbaGaem4zaCgabeaaaaa@36E1@ where *sd*_*g *_represents the standard deviation of the ratios measured from the genomic features. In nine experimental hybridizations the standard deviation of the ratios of genes equally transcribed was greater, from 1.6 to 2.4 fold, if measured in ORFs features than if measured in genomic features. For this reason, *b *was estimated assuming that the standard deviation of the ratios of the genes equally transcribed is twice as much as that of the ratios measured from genomic features.

After data standardization, the ratios of equally transcribed genes are expected to be centred on 0. Thus, the centred value, *a*_0_, of the hyperparameter *A *is estimated by the method of moments as a^0=0−0.5772⋅b^
 MathType@MTEF@5@5@+=feaafiart1ev1aqatCvAUfKttLearuWrP9MDH5MBPbIqV92AaeXatLxBI9gBaebbnrfifHhDYfgasaacPC6xNi=xH8viVGI8Gi=hEeeu0xXdbba9frFj0xb9qqpG0dXdb9aspeI8k8fiI+fsY=rqGqVepae9pg0db9vqaiVgFr0xfr=xfr=xc9adbaqaaeGacaGaaiaabeqaaeqabiWaaaGcbaGafmyyaeMbaKaadaWgaaWcbaGaeGimaadabeaakiabg2da9iabicdaWiabgkHiTiabicdaWiabc6caUiabiwda1iabiEda3iabiEda3iabikdaYiabgwSixlqbdkgaIzaajaaaaa@3A8F@

To estimate the parameters *α *and *β*, the first consideration is that for the genomic features *p*_0 _= *p*_1 _= 0.5. Thus, the prior distribution of *A *for the genomic features can be simplified as

ξA prior(a)=1bβαΓ(α)eabαe−βeab−∞<a<∞
 MathType@MTEF@5@5@+=feaafiart1ev1aqatCvAUfKttLearuWrP9MDH5MBPbIqV92AaeXatLxBI9gBaebbnrfifHhDYfgasaacPC6xNi=xI8qiVKYPFjYdHaVhbbf9v8qqaqFr0xc9vqFj0dXdbba91qpepeI8k8fiI+fsY=rqGqVepae9pg0db9vqaiVgFr0xfr=xfr=xc9adbaqaaeGacaGaaiaabeqaaeqabiWaaaGcbaqbaeqabeGaaaqaaGGaciab=57a4naaBaaaleaacqWGbbqqcqqGGaaicqWGWbaCcqWGYbGCcqWGPbqAcqWGVbWBcqWGYbGCaeqaaOGaeiikaGIaemyyaeMaeiykaKIaeyypa0tcfa4aaSaaaeaacqaIXaqmaeaacqWGIbGyaaWaaSaaaeaacqWFYoGydaahaaqabeaacqWFXoqyaaaabaGaeu4KdCKaeiikaGIae8xSdeMaeiykaKcaaOGaemyzau2aaWbaaSqabeaajuaGdaWcaaqaaiabdggaHbqaaiabdkgaIbaaliab=f7aHbaakiabdwgaLnaaCaaaleqabaGaeyOeI0Iae8NSdiMaemyzau2aaWbaaWqabKqbagaadaWcaaqaaiabdggaHbqaaiabdkgaIbaaaaaaaaGcbaaccaGae4NeI0IaeyOhIuQaeyipaWJaemyyaeMaeyipaWJaeyOhIukaaaaa@5C5D@

As indicated in the Appendix, the transformation X=eAb
 MathType@MTEF@5@5@+=feaafiart1ev1aqatCvAUfKttLearuWrP9MDH5MBPbIqV92AaeXatLxBI9gBaebbnrfifHhDYfgasaacPC6xNi=xH8viVGI8Gi=hEeeu0xXdbba9frFj0xb9qqpG0dXdb9aspeI8k8fiI+fsY=rqGqVepae9pg0db9vqaiVgFr0xfr=xfr=xc9adbaqaaeGacaGaaiaabeqaaeqabiWaaaGcbaGaemiwaGLaeyypa0Jaemyzau2aaWbaaSqabKqbagaadaWcaaqaaiabdgeabbqaaiabdkgaIbaaaaaaaa@3289@ has a gamma distribution with shape parameter *α *and scale parameter 1/*β*. Accordingly, the ratios measured from the genomic features, *r*_*g*_, are transformed into rg′=erg−0.5772bb
 MathType@MTEF@5@5@+=feaafiart1ev1aaatCvAUfKttLearuWrP9MDH5MBPbIqV92AaeXatLxBI9gBaebbnrfifHhDYfgasaacPC6xNi=xH8viVGI8Gi=hEeeu0xXdbba9frFj0xb9qqpG0dXdb9aspeI8k8fiI+fsY=rqGqVepae9pg0db9vqaiVgFr0xfr=xfr=xc9adbaqaaeGacaGaaiaabeqaaeqabiWaaaGcbaGaemOCai3aa0baaSqaaiabdEgaNbqaaOGamai4gkdiIcaacqGH9aqpcqWGLbqzjuaGdaahaaqabeaadaWcaaqaaiabdkhaYnaaBaaabaGaem4zaCgabeaacqGHsislcqaIWaamcqGGUaGlcqaI1aqncqaI3aWncqaI3aWncqaIYaGmcqWGIbGyaeaacqWGIbGyaaaaaaaa@40EF@. From the transformed ratios, the maximum likelihood estimates for the parameters *α *and *α *of a gamma distribution are obtained as described by [15]. For the gamma function we used the approximation derived by Lanczos [16].

To estimate the expected value and variance of *A *(see Appendix), the digamma function was approximated by using the formula 6.3.16 p.259 of [17].

Table 1 shows the estimations for the three datasets.

**Table 1:** Estimates from the 1 normalized datasets for ArrayLeaRNA approach

Parameters and Statistics	Dataset I	Dataset II	Dataset III
Average of ^2^*R *from genomic features	0	0	0
Standard deviation of *R *from genomic features	0.566	0.244	0.566
Parameter *b *of the Gumbel distribution for *R*	0.882	0.381	0.883
Parameter *α *of the prior distribution for *A*	1.65	4.15	2.87
Parameter *β *of the prior distribution for *A*	2.11	6.51	4.26
Prior expected value for *A*	-0.509	-0.220	-0.509
Prior variance for *A*	0.614	0.0393	0.318
Parameter *α *of the ^3^posterior distribution for *A*	7.65	8.15	3.87
Parameter *β *of the posterior distribution for *A*	8.11	10.5	5.26
Posterior expected value for *A*	-0.110	-0.121	-0.389
Posterior variance for *A*	0.150	0.0189	0.122

^1 ^Normalization is based on the genomic features measurements; ^2 ^Logarithm to the base 2 of the ratio between the intensities; ^3 ^Assuming *p*_1_= *p*_0 _= 0.5, *R *= 0 and 6, 4 and 1 replicates for Datasets I, II and III, respectively.

The values of *p*_0 _and *p*_1 _are calculated from the transcription pattern of the operon to which the gene in study belongs. If the operon information is not available, *p*_0 _= *p*_1_= 0.5. But if available, *p*_0 _and *p*_1 _are calculated by iterating the analysis and could affect the posterior probability *P *(equation 4) by taking values different from 0.5. The greater the value of *p*_0_, the greater is *P*, while the greater the value of *p*_1_, the greater is the chance of that gene being differentially expressed. Initially, *p*_0 _and *p*_1 _do not favour any result with values *p*_0 _= *p*_1 _= 0.5. With these initial values, the hybridization dataset is analyzed for the first time and a preliminary classification is obtained for each gene in the dataset. Genes are assumed to have increased or decreased expression relative to the control sample (i.e. up or down regulated) when *P *(*a *≥ *a*_0_) < 0.01. The values of *p*_0 _and *p*_1 _are updated as follows:

p0=12+(T−1)N04(T+1)T−(T−1)N14(T+1)Tp1=12−(T−1)N04(T+1)T+(T−1)N14(T+1)T
 MathType@MTEF@5@5@+=feaafiart1ev1aaatCvAUfKttLearuWrP9MDH5MBPbIqV92AaeXatLxBI9gBaebbnrfifHhDYfgasaacPC6xNi=xI8qiVKYPFjYdHaVhbbf9v8qqaqFr0xc9vqFj0dXdbba91qpepeI8k8fiI+fsY=rqGqVepae9pg0db9vqaiVgFr0xfr=xfr=xc9adbaqaaeGacaGaaiaabeqaaeqabiWaaaGcbaqbaeqabiqaaaqaaiabdchaWnaaBaaaleaacqaIWaamaeqaaOGaeyypa0tcfa4aaSaaaeaacqaIXaqmaeaacqaIYaGmaaGccqGHRaWkjuaGdaWcaaqaaiabcIcaOiabdsfaujabgkHiTiabigdaXiabcMcaPiabd6eaonaaBaaabaGaeGimaadabeaaaeaacqaI0aancqGGOaakcqWGubavcqGHRaWkcqaIXaqmcqGGPaqkcqWGubavaaGccqGHsisljuaGdaWcaaqaaiabcIcaOiabdsfaujabgkHiTiabigdaXiabcMcaPiabd6eaonaaBaaabaGaeGymaedabeaaaeaacqaI0aancqGGOaakcqWGubavcqGHRaWkcqaIXaqmcqGGPaqkcqWGubavaaaakeaacqWGWbaCdaWgaaWcbaGaeGymaedabeaakiabg2da9KqbaoaalaaabaGaeGymaedabaGaeGOmaidaaOGaeyOeI0scfa4aaSaaaeaacqGGOaakcqWGubavcqGHsislcqaIXaqmcqGGPaqkcqWGobGtdaWgaaqaaiabicdaWaqabaaabaGaeGinaqJaeiikaGIaemivaqLaey4kaSIaeGymaeJaeiykaKIaemivaqfaaOGaey4kaSscfa4aaSaaaeaacqGGOaakcqWGubavcqGHsislcqaIXaqmcqGGPaqkcqWGobGtdaWgaaqaaiabigdaXaqabaaabaGaeGinaqJaeiikaGIaemivaqLaey4kaSIaeGymaeJaeiykaKIaemivaqfaaaaaaaa@75E2@

where *N*_0 _and *N*_1 _depends on the classification of the gene in study. When the gene in study is equally transcribed, *N*_0 _= Card (genes equally transcribed) and *N*_1 _= |Card (genes up-regulated) – Card (genes down-regulated)| where Card (.) denotes the cardinal number of the set of genes. If some genes of the operon are up-regulated and some of them are down-regulated in the same sample they cancel each other and do not decrease the probability of the equal transcription in both samples; hence, *N*_1 _is calculated as the absolute value of the difference between the cardinal numbers of the up and down regulated genes. If the gene in study is classified as up-regulated, *N*_0 _= Card (genes equally transcribed) + Card (genes down-regulated) and *N*_1 _= Card (genes up-regulated). If the gene in study is down-regulated, *N*_0 _= Card (genes equally transcribed) + Card (genes up-regulated) and *N*_1 _= Card (genes down-regulated). *T *is the number of genes of the operon involved in the estimation, i.e. *T *= *N*_1 _+ *N*_2 _+ 1. Therefore, *p*_1 _and *p*_0 _are based only on those genes successfully analysed within an operon. The operons composition is that published at [18-20]. The analysis of the dataset is iterated several times. Five iterations have been proven sufficient. In each iteration genes are reclassified and the values of *p*_0 _and *p*_1 _are updated.

## Results

We demonstrate a Bayesian method of microarray data analysis based on using internal positive controls for normalisation and as a basis of the Bayesian model, plus using predicted operon structure to improve assignments of differentially expressed genes. The method has been tested on microarray data obtained from three different bacterial organisms.

**Figure 1:**
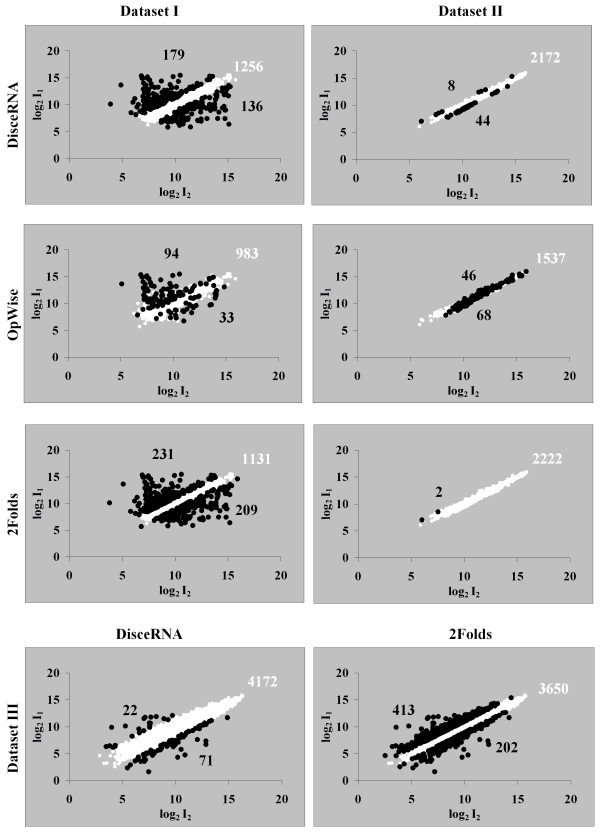
**Comparison of gene expression analysis approaches on experimental datasets**. Analysis of the three datasets with the three approaches: ArrayLeaRNA after normalization based on genomic controls and two fold cut-off and OpWise after LOWESS normalization. Genes are classified as equally expressed (○), and up or down-regulated (●). Number of genes is reported for each category. The cut-off values for the posterior probability of equal expression were 0.01 for ArrayLeaRNA and 0.99 and 0.01 for OpWise.

Fig 1 shows the results for the analysis of the three datasets. Dataset I is the result of a microarray hybridization prepared from two differentially labelled cDNA samples obtained from two strains of *C. jejuni*, 81116 and NCTC 11168. Amplicons specific to strain 81116 (138) should only hybridize labelled cDNA from this strain and should therefore represent a set of down-regulated genes in the strain NCTC 11168. Likewise for amplicons specific to strain NCTC 11168 (134). Dataset II results from two samples of differentially labelled cDNA obtained from two replicated cultures of *S. pneumoniae *TIGR4 and hybridized on a single slide. Dataset III was prepared from two samples of cDNA obtained from two replicated cultures of *E. coli *K12 but the samples of cDNA were mixed with labelled genomic DNA and hybridized independently on 2 microarray slides. The dataset includes the intensities measured for the cDNA samples only. Datasets II and III were obtained from replicated cultures and all the genes are expected to be equally expressed. We expect these datasets to show different variability, which will be reflected in the genomic control measurements, as a result of the different hybridization protocols.

**Figure 2:**
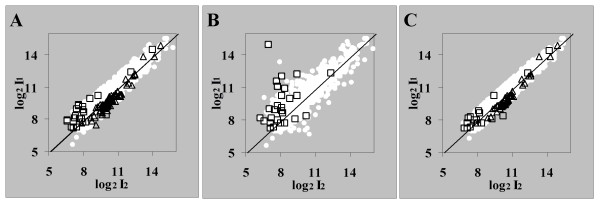
**Misclassifications in dataset I**. Misclassified genes in dataset I by ArrayLeaRNA (A), OpWise (B) and the two fold cut-off (C). The features unique to the strains NCTC 81116 (△) and NCTC 11168 (□) and misclassified as equally described by ArrayLeaRNA and the two fold cut-off showed intensities typical of equally transcribed genes. Only features unique to the strains NCTC 11168 (□) could be analysed by OpWise. The features classified as equally expressed (○) are also shown for each approach. The cut-off values for the posterior probability of equal expression were 0.01 for ArrayLeaRNA and 0.99 and 0.01 for OpWise.

For dataset I ArrayLeaRNA classified 315 differentially expressed genes. From the features unique to the strain 81116, ArrayLeaRNA misclassified 69 ORFs while 24 misclassifications were detected from the features unique to the strain NCTC 11168. As shown in Fig 2, the intensities from those misclassified features showed typical values of equally expressed genes. In dataset II, ArrayLeaRNA misclassified 52 genes in dataset II and 93 genes in dataset III. Some of these misclassified genes showed large ratios and may reflect real changes in gene expression due to subtle experimental differences during bacterial culture or technical microarray artefacts.

The OpWise method was applied by using the computing tool provided by the authors. The cut-off values for the posterior probability of equal expression were 0.99 and 0.01 for OpWise. This is equivalent to the cut off chosen for ArrayLeaRNA that is 0.01 of posterior probability associated to the absolute value of the ratio between intensities.

In dataset I, the 138 ORFs unique to strain 81116 could not be analysed because the operon composition is not available for this strain. Regarding the features unique to the strain NCTC 11168, 26 features were misclassified as equally expressed. Some of the misclassified features showed large ratios (Fig 2). The misclassification of features with large rations was associated to features with few replicate measurements as a result of discarding bad quality measurements. For dataset I, OpWise was too conservative and failed to classify clearly differentially expressed genes (Fig 1). Opwise is a Bayesian approach based on a Gaussian model that can be expressed in terms of the *t *distribution. The posterior probability can be formulated without taking into account the operon information. When the model underlying OpWise was applied without operon information, only 33 genes, with ratios greater than 4.3, were classified as differentially expressed and the number of features unique to the strain NCTC 11168 that were misclassified as equally expressed increased to 41. Therefore, for this dataset, the operon information decreased the number of misclassified genes. In dataset II, OpWise misclassified 114 genes that showed equal expression in both samples. In this case, the model without operon information performed better and did not misclassify any gene. Dataset III could not be analysed with OpWise because there was only one replicate of each ORF.

When applying the two-fold cut-off value in dataset I, 440 genes showed ratios greater than the arbitrarily chosen two-fold value; this is significantly higher than the 315 and the 86 genes differentially expressed according to ArrayLeaRNA and OpWise methods, respectively. Fifty five features unique to 81116 and 17 features unique to 11168 were incorrectly classified but as mentioned above and shown in Fig 2, these features exhibited typical values of identically expressed genes. The two-fold approach did not misclassify any gene on dataset II but misclassified 615 genes in dataset III. The variability of dataset II was relatively small because the mixture of differentially labelled cDNA was hybridized on a single slide and each ORF had ca. 6 replicates. This contrasts the set-up of the experiment from which dataset III was derived, where the labelled cDNA samples were mixed with labelled genomic DNA and hybridized on different slides and the final dataset contained only 1 ratio per ORF. Therefore variability of dataset III was greater and 615 genes of this dataset showed ratios larger than 2. A constant cut-off value arbitrarily chosen is not an advisable analysis technique in any case.

**Figure 3:**
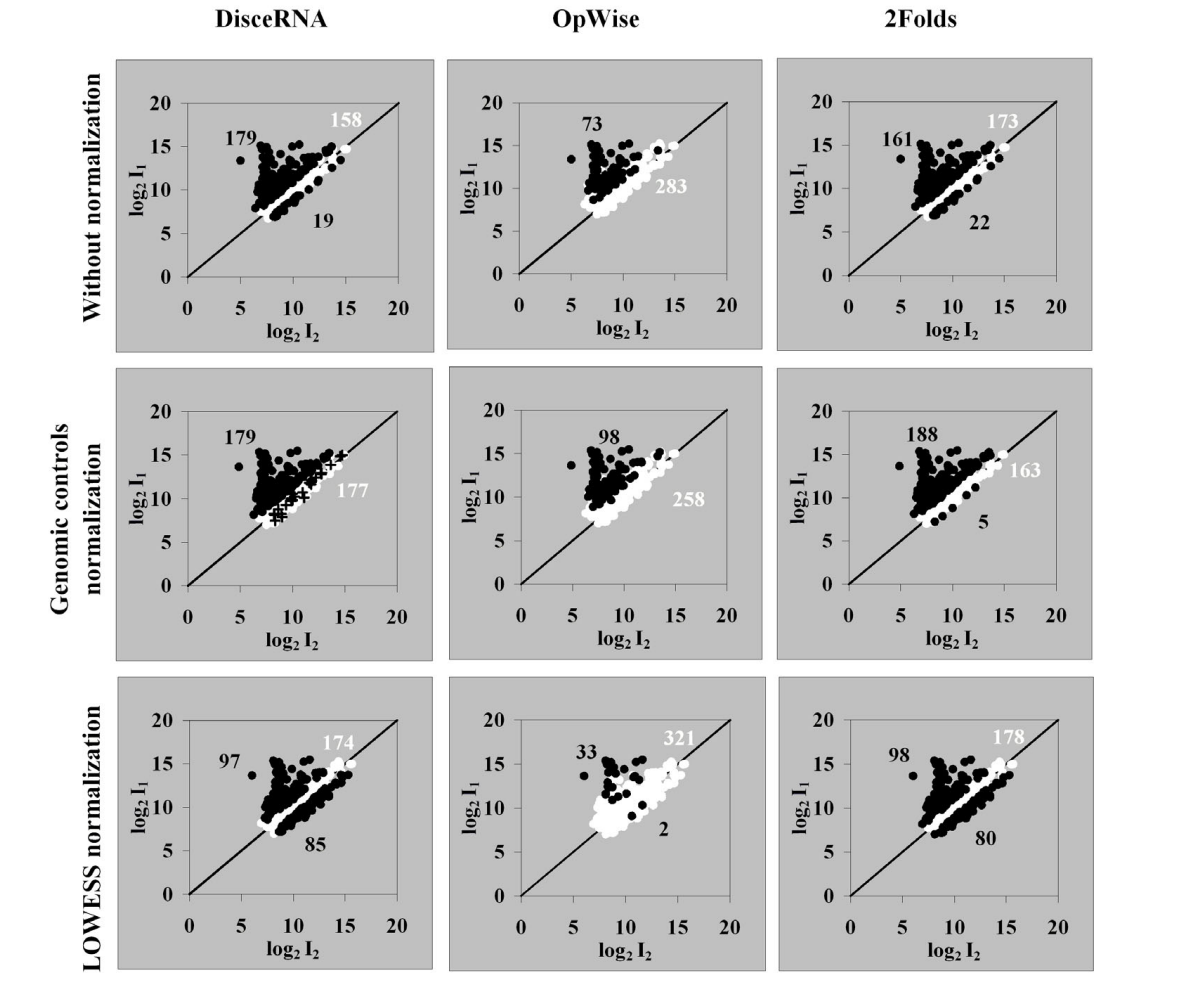
**Effect of the normalization and analysis approach on dataset IV**. Effect of the normalization and analysis approach on the results on gene expression derived from dataset IV which includes 178 genes up-regulated in sample 1 and 178 genes equally transcribed. Genes are classified as equally expressed (○), and up or down-regulated (●). Number of genes is reported for each category. Crosses (+) show the intensities of genomic controls. The cut-off values for the posterior probability of equal expression were 0.01 for ArrayLeaRNA and 0.99 and 0.01 for OpWise.

ArrayLeaRNA was applied after normalizing the datasets with the correcting factors estimated from the genomic features. OpWise and the two-fold cut-off were applied after LOWESS normalization. The effect of the normalization technique was studied in Dataset IV which was generated from Dataset I as described above. Dataset IV is an asymmetric gene expression dataset with 178 up-regulated genes in one of the samples and 178 equally transcribed in both samples. The mean ratio of the whole dataset is very different from the mean ratio of the equally transcribed genes. Dataset IV was analysed using ArrayLeaRNA, OpWise, and the two-fold cut-off following a) normalization using the genomic controls, b) LOWESS normalization (according to the LOWESS regression scores estimated for this sub-dataset), and c) without using any normalization method, i.e. non-transformed data (Fig 3). When applying ArrayLeaRNA, the parameters of the model were always estimated from the original genomic features and therefore equal for all the cases. In the dataset normalized according to the genomic controls, the performance of the three approaches improved with respect to the non transformed dataset. However, after LOWESS normalization all methods misclassified a large number of genes. OpWise misclassified 153 up-regulated genes in sample 1 as equally transcribed; the two-fold approach misclassified 80 genes as up-regulated in sample 2 and 80 genes as equally transcribed. ArrayLeaRNA misclassified 85 genes as up-regulated in sample 2 and 81 as equally transcribed. When applying the normalization approach based on the genomic features, the genomic features and the identically expressed genes showed the same average ratios, so that the normalized ratios of the equally transcribed genes were centred in 1 and accordingly the ratios of the differentially expressed genes were corrected, therefore, results improved with all the analysis approaches. With LOWESS normalization, the centre of gravity of the whole dataset was considered to be the centre of gravity of the equally transcribed genes. Thus the normalized ratios of the genes located in the centre of gravity of the whole dataset were close to 1 and wrongly considered to be equally expressed. On the other hand, the ratios of the truly equally transcribed genes were shifted towards typical values of differentially transcribed genes. Hence, after this normalization approach, misclassification is due to occur with any kind of analysis. Other problems derived from LOWESS normalization are described in [21].

In the non-transformed and normalized with genomic controls datasets, ArrayLeaRNA was more accurate than the other two approaches. In the non-transformed dataset, ArrayLeaRNA misclassified 19 genes and only 1 in the dataset normalized according to the genomic controls. Some genes equally transcribed showed ratios slightly greater than 2, therefore, the two-fold approach misclassified 39 genes in the non-transformed dataset and 15 after genomic normalization. OpWise misclassified 105 and 80 genes in the non-transformed and normalized dataset, respectively. OpWise misclassified a large number of genes up-regulated in sample 1 as equally transcribed. The two-fold cut-off approach with narrower boundaries mostly misclassified genes equally transcribed as up-regulated (Fig 3).

## Discussion

We present a Bayesian microarray analysis tool which takes advantage of genomic DNA control features and predicted operon structure to provide an accurate and informed analysis of transcriptomic data.

The estimation of the model parameters relies on the ratios measured from genomic DNA features of the appropriate strain(s) printed on the microarray slide. This is the scenario for which ArrayLeaRNA has been designed. If genomic DNA features are not printed on the slide, the computing tool implementing ArrayLeaRNA allows the user to input the ratio at the boundary between genes equally and differentially expressed. The parameters of the model are then estimated to obtain approximately the desired boundary. An alternative solution could be to identify genes shown to have consistently non-changing expression patterns instead of printed genomic DNA

**Figure 4:**
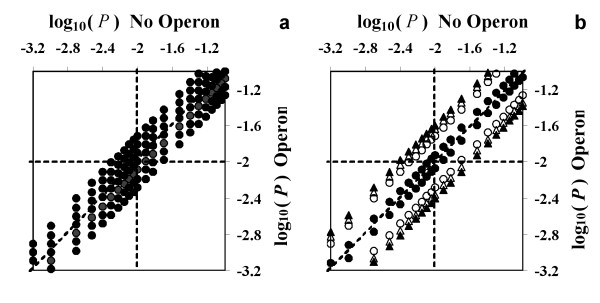
**Relationship between the probability of identical expression in both samples, *P*, without operon information and with operon information. **Dashed lines represent the diagonal of equality and the value of *P *= 0.01 in both axes.(a) Effect of the transcriptional pattern on *P*. The plot represents the case in which the gene in study is up-regulated in sample 1 and belongs to an operon with 6 more genes from which 0, 1, 2, 3, 4, 5 or 6 are up-regulated in sample 1 (lines from top to bottom). (b) Effect of the operon size on *P*. The most extreme transcriptional pattern is represented: the gene in study is temporally classified as equally (or differentially) transcribed and the rest of the genes in the operon are classified as differentially (or equally) transcribed. Different operon sizes, 1 (●), 6 (0), 12 (△) and 24 (▲) genes, are considered.

ArrayLeaRNA can be run without knowing the operon structure of the genome. Operon predictions are not indispensable for the analysis but help the classification of genes with low confidence measurements. The operon information is quantified by *p*_0 _and *p*_1_, which comply with *p*_0 _+ *p*_1 _= 1. The greater *p*_0_, the greater is the probability that the gene in study is identically expressed in both samples. The value of *p*_0 _is greater than 0.5 if the number of equally expressed genes in the operon is higher than the number of differentially expressed genes. The initial value of *p*_0 _and *p*_1 _is 0.5 and has no effect on *P*. Moreover, *P *is very robust to the prior values of these two parameters, even if these values were completely erroneous, the genes classified with high confidence would not be affected. Starting from the initial values, the analysis is iterated to obtain the true values for *p*_0 _and *p*_1_, and recalculate *P *to help correctly classify genes with low confidence measurements. The values of *p*_0 _and *p*_1_, and thus *P*, depends on both the predicted structure of the operon and the number of genes of the operon. Fig 4a shows the effect of the transcription pattern on *P *by using an example in which the gene in study is up-regulated in one sample and belongs to an operon with 6 additional genes. If all the other genes of the operon are also up-regulated in that sample then *P *decreases by ca. two-fold, while if they are equally transcribed *P *increases by ca. two-fold; if 3 genes of the operon are up-regulated and 3 equally transcribed, *P *is not affected. The same behaviour is observed if the gene in study is classified as equally transcribed. That means that only genes with values for *P *very close to the cut-off values, i.e. low confidence measurements, are reclassified. Fig 4b shows the effect of the size of the operon on *P*. The plot represents the transcriptional pattern with the greatest effect on *P*: the gene in study is temporally classified as equally (or differentially) transcribed and the rest of the genes in the operon have the opposite classification. When the operon has only 2 genes, *P *remains largely unaffected. The effect of the transcriptional pattern on *P *increases when the size of the operon increases, but it soon converges so that an operon of 12 genes has practically the same effect as an operon of 25 genes. We chose this range of numbers because we observed that the largest predicted operons for *E. coli *K12, *C. jejuni *NCTC 11168 and *S. pneumoniae *TIGR4 contain, 28, 24 and 15 genes, respectively. In any case, *P *is slightly affected so that the classification of genes truly expressed either in one or both samples is not modified; for instance, for an operon with 25 genes, only genes with values for *P *greater than 0.003 would be reclassified as equally transcribed (assuming *P *= 0.01 as a cut off) and only genes with values for *P *smaller than 0.03 would be reclassified as differentially expressed. ArrayLeaRNA differs from OpWise in this respect. OpWise does not take into account the size of the operon but uses only the information of the pair of genes flanking the gene in study. With ArrayLeaRNA, only genes with low confidence measurements, showing ratios close to the boundary between equally and differentially transcribed genes, may be reclassified. Thus reclassifications, due to the use of the operon information, are not possible for genes with high confidence measurements. In contrast, when taking into account the operon information, OpWise misclassified 114 genes as differentially expressed in dataset II although many of them had ratios with values of ca. 1 (Fig 1). This is a consequence of the fact that OpWise uses the transcriptional information of only the two genes flanking the gene in study and in Dataset II these genes had very similar average ratios and relatively small variances. Thus, several genes with average ratios even smaller than 1.1 were incorrectly classified as differentially expressed.

The underlying model in ArrayLeaRNA is different from the Gaussian models developed by [4-8]. The approaches of [4,5,7] model the expression measurements by normal distributions parameterized by means and variances with conjugate prior distributions and assuming dependence between means and variance. As an alternative to full Bayesian treatment, [4] suggested the use of an intermediate solution using a regularized t-test, in which the variance is replaced by the posterior mean of the variance of the model. [6] modified the Gaussian model by introducing a Bernoulli random variable, indicating whether the gene is differentially expressed. The parameter, *p*, of the Bernoulli distribution describes the proportion of differentially expressed genes and it is estimated by the iteration of the analysis. The effect of this parameter in the posterior probability is similar to that of *p*_0_, or *p*_1_, introduced in ArrayLeaRNA to quantify the operon information. The model underlying OpWise [8] is also based in the above Gaussian model but introducing a new component of the error called systematic error or bias estimated from the measurements of the genes belonging to the same operon. The posterior probabilities are estimated for single genes without operon information and with operon information as a mixture of the posterior probabilities of all the possible operon composition combinations of the gene in study with the pair of genes adjacent to it.

**Figure 5:**
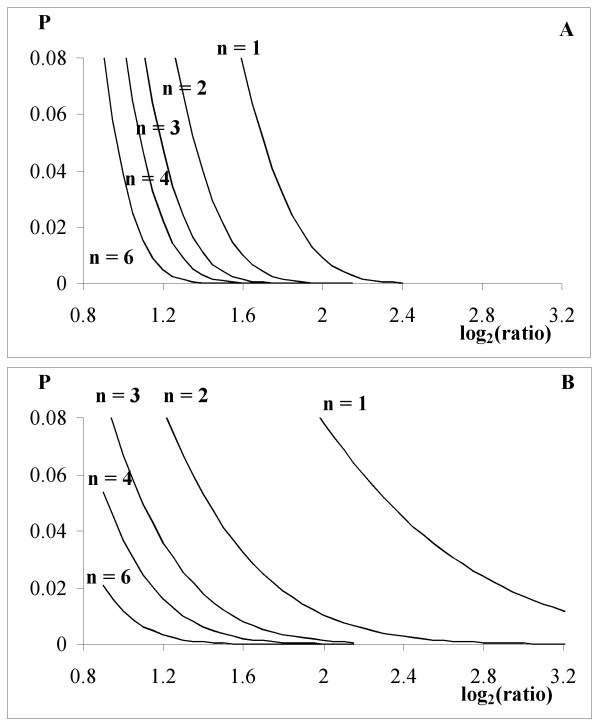
**Effect of the assumed distribution, A) Gumbel or B) Gaussian, for the log of the ratio and of the number of replicates on the posterior probability of identical expression, *P*. **The plots represent the case in which the expected value of the log_2 _(ratio) for genes identically transcribed in both samples is 0 and their variance equal to 1. The number of replicates (n) varies from 1 to 6.

The model underlying ArrayLeaRNA assumes that the log ratios have a Gumbel distribution with a known scale parameter, a location parameter with conjugate prior and the genes being independent. The probability of equal transcription is estimated in the steepest tail of the posterior distribution, which results in a steeper probability curve and a neater boundary between genes equally and differentially expressed than when using the Gaussian model (Fig 5). The number of replicates affects the posterior probability but this effect is smaller when using the Gumbel distribution. ArrayLeaRNA gives more conclusive results with very low number of replicates than the Gaussian approach (Fig 5). For the case presented in Fig 5, the log ratio for which the probability of equal expression is less than 0.01 varies between 1.35, if n = 6, and 2, if n = 1. As expected in statistical inference, replication leads to greater resolution of small differences in gene expression [22]. In general, statistical analysis techniques are conservative with small sample sizes and may under estimate the number of genes up- or down-regulated. Researchers should seek confirmation of results before proceeding to undertake more elaborate, gene-specific experiments.

The scale parameter of the Gumbel distribution is estimated from the standard deviation of the ratios measured from the genomic control features. This is an estimation of the variability in equally expressed genes intrinsic to the experimental hybridization and overcomes the uncertainty of estimations with low number of replicates and the fact that replicated measurements, in the same or replicated slides and from the same or replicated experiments, may not reflect the variability of the set of features with equal amounts of hybridized transcripts from each sample. Thus the genomic DNA features printed on the microarray slide offer a significant advantage not only for data normalization but also for determining whether the differences in expression are significant based on the robust estimation of the variability of equally transcribed genes. Moreover, printed genomic DNA offers distinct advantages over other types of features designed to be non-crosshybridizing controls (i.e. yeast ORFs in bacterial microarrays) in combination with exogenously added cDNA. Such controls will only account for part of the experimental variability, compared to printed genomic DNA, which reflects the variability arising from the experimental hybridization of the labelled cDNA prepared from the RNA under study.

## Conclusion

We have introduced a Bayesian model based on the Gumbel distribution, in combination with printed genomic DNA controls and predicted operon information, and demonstrated that it is a robust method for analysing microarray expression profiles. The method is applicable to data derived from hybridizations of labelled cDNA samples as well as from hybridizations of labelled cDNA with genomic DNA. The method can equally be applied to datasets where differentially regulated genes predominate. The method we introduce performed better than two existing methods (OpWise and the two-fold cut-off) when analysing the experimental datasets presented in this work.

## Methods

### Bacterial Strains

*Campylobacter jejuni *strains NCTC 11168 and 81116 (NCTC 11828) were grown at 37°C under microaerophilic conditions (10% CO_2_, 5% O_2_, 85% N_2_; relative humidity 80%) on Skirrow agar plates or in Brucella broth using a MACS-MG-1000 controlled atmosphere workstation (DW Scientific, UK).

*Streptococcus pneumoniae *JNR7/87 (also called TIGR4) was grown at 37°C in tryptone soy broth or on tryptone soy agar plates supplemented with 5% horse blood.

*Escherichia coli *K-12 strain MG1655 was grown at 25°C in Luria-Bertani broth (10 g/l Tryptone, 5 g/l yeast extract and 10 g/l NaCl; pH 7.2) with 0.2% glucose.

### Construction of DNA microarrays

Internal DNA fragments corresponding to unique segments of each open reading frame (ORFs) annotated in the genome of the strain were PCR-amplified using gene-specific primers. DNA probes and various concentrations of chromosomal DNA were spotted on GAPS II slides (Corning) using a in-house Stanford designed arrayer and the recommended software and protocols [23].

The following three DNA microarrays were used: 1. A microarray representing six replicates of all ORFs from *C. jejuni *NCTC 11168 and 138 ORFs unique to strain 81116. From the ORFs of *C. jejuni *NCTC 11168, 134 are missing genes in the strain 81116; 2. A microarray representing four replicates of all open reading frames from the *S. pneumoniae *TIGR4 [24]; 3. A microarray representing one replicate of all open reading frames from *E. coli *K-12 MG1655 [25].

All the arrays contained ca.100 features of serially diluted chromosomal DNA (ca. 15–20 replicates of each dilution) isolated from the reference strain(s) used to construct the array. These features are referred as genomic controls and they are used in data standardization and in data analysis.

### RNA and DNA purification and microarray hybridizations

RNA was purified from *S. pneumoniae *as described in [26]; RNA was purified from *E. coli *as described in [27]; RNA was purified from *C. jejuni *as described in [28]. RNA quality and quantity was checked using the Agilent 2100 Bioanalyzer [29]. DNA was isolated from bacteria using the QIAgen DNeasy™ method (QIAgen)

cDNA was prepared from RNA using Stratascript RT (Stratagene) and labelled with Cy3-dCTP and Cy5-dCTP (Amersham). Labelled cDNA and DNA were purified using the QIAquick PCR purification kit (QIAgen). Differentially labelled cDNA or cDNA and DNA were mixed and hybridized on a microarray slide at 62°C overnight. Following hybridization, microarray slides were washed and scanned using an Axon GenePix 4000A microarray laser scanner (Axon Instruments, CA) and the feature data generated using GenePix Pro software (Molecular Devices). The fluorescence intensity was defined as the median of the foreground intensities in each feature with the median background subtracted.

## Availability and requirements

ArrayLeaRNA is implemented in Visual Basic and freely available as an Excel add-in at . The user requires Excel 2000 or later versions installed in their computer.

## Authors' contributions

CP and MR contributed equally to this work

## Appendix

Conjugate family of distributions for the location parameter of the Gumbel distribution

Let *R *follow a Gumbel distribution with density function

gR(r)=1beA−rbe−eA−rb−∞<r<∞
 MathType@MTEF@5@5@+=feaafiart1ev1aqatCvAUfKttLearuWrP9MDH5MBPbIqV92AaeXatLxBI9gBaebbnrfifHhDYfgasaacPC6xNi=xI8qiVKYPFjYdHaVhbbf9v8qqaqFr0xc9vqFj0dXdbba91qpepeI8k8fiI+fsY=rqGqVepae9pg0db9vqaiVgFr0xfr=xfr=xc9adbaqaaeGacaGaaiaabeqaaeqabiWaaaGcbaqbaeqabeGaaaqaaiabdEgaNnaaBaaaleaacqWGsbGuaeqaaOGaeiikaGIaemOCaiNaeiykaKIaeyypa0tcfa4aaSaaaeaacqaIXaqmaeaacqWGIbGyaaGccqWGLbqzdaahaaWcbeqcfayaamaalaaabaGaemyqaeKaeyOeI0IaemOCaihabaGaemOyaigaaaaakiabdwgaLnaaCaaaleqabaGaeyOeI0Iaemyzau2aaWbaaWqabKqbagaadaWcaaqaaiabdgeabjabgkHiTiabdkhaYbqaaiabdkgaIbaaaaaaaaGcbaaccaGae8NeI0IaeyOhIuQaeyipaWJaemOCaiNaeyipaWJaeyOhIukaaaaa@4D5C@

where *A *and *b *are the location and scale parameters, respectively.

The density function for a simple random sample of *R *with *n *independent measurements, *r*_1_..*r*_*n*_, is:

gR(r1..rn)=1bnenAbe−∑i=1nribe−keAb
 MathType@MTEF@5@5@+=feaafiart1ev1aqatCvAUfKttLearuWrP9MDH5MBPbIqV92AaeXatLxBI9gBaebbnrfifHhDYfgasaacPC6xNi=xI8qiVKYPFjYdHaVhbbf9v8qqaqFr0xc9vqFj0dXdbba91qpepeI8k8fiI+fsY=rqGqVepae9pg0db9vqaiVgFr0xfr=xfr=xc9adbaqaaeGacaGaaiaabeqaaeqabiWaaaGcbaGaem4zaC2aaSbaaSqaaiabdkfasbqabaGccqGGOaakcqWGYbGCdaWgaaWcbaGaeGymaedabeaakiabc6caUiabc6caUiabdkhaYnaaBaaaleaacqWGUbGBaeqaaOGaeiykaKIaeyypa0tcfa4aaSaaaeaacqaIXaqmaeaacqWGIbGydaahaaqabeaacqWGUbGBaaaaaOGaemyzau2aaWbaaSqabKqbagaadaWcaaqaaiabd6gaUjabdgeabbqaaiabdkgaIbaaaaGccqWGLbqzdaahaaWcbeqaaiabgkHiTmaaqahajuaGbaWaaSaaaeaacqWGYbGCdaWgaaqaaiabdMgaPbqabaaabaGaemOyaigaaaadbaGaemyAaKMaeyypa0JaeGymaedabaGaemOBa4gaoiabggHiLdaaaOGaemyzau2aaWbaaSqabeaacqGHsislcqWGRbWAcqWGLbqzdaahaaadbeqcfayaamaalaaabaGaemyqaeeabaGaemOyaigaaaaaaaaaaa@59D0@

It can be shown that k=∑i=1ne−rib
 MathType@MTEF@5@5@+=feaafiart1ev1aqatCvAUfKttLearuWrP9MDH5MBPbIqV92AaeXatLxBI9gBaebbnrfifHhDYfgasaacPC6xNi=xH8viVGI8Gi=hEeeu0xXdbba9frFj0xb9qqpG0dXdb9aspeI8k8fiI+fsY=rqGqVepae9pg0db9vqaiVgFr0xfr=xfr=xc9adbaqaaeGacaGaaiaabeqaaeqabiWaaaGcbaGaem4AaSMaeyypa0ZaaabCaeaacqWGLbqzdaahaaWcbeqaaiabgkHiTKqbaoaalaaabaGaemOCai3aaSbaaeaacqWGPbqAaeqaaaqaaiabdkgaIbaaaaaaleaacqWGPbqAcqGH9aqpcqaIXaqmaeaacqWGUbGBa0GaeyyeIuoaaaa@3C72@ is a sufficient statistic for the Gumbel distribution

When the scale parameter, *b*, is known, the Gumbel distribution belongs to the exponential family and a conjugate family of distributions exists for the parameter *A*, i.e. the prior and posterior distributions of *A *differ only in the value of a finite parameter vector. A conjugate family of distributions for the parameter *A *of the Gumbel distribution is given by:

fA(a)=1bβαΓ(α)eabαe−βeab−∞<a<∞
 MathType@MTEF@5@5@+=feaafiart1ev1aqatCvAUfKttLearuWrP9MDH5MBPbIqV92AaeXatLxBI9gBaebbnrfifHhDYfgasaacPC6xNi=xI8qiVKYPFjYdHaVhbbf9v8qqaqFr0xc9vqFj0dXdbba91qpepeI8k8fiI+fsY=rqGqVepae9pg0db9vqaiVgFr0xfr=xfr=xc9adbaqaaeGacaGaaiaabeqaaeqabiWaaaGcbaqbaeqabeGaaaqaaiabdAgaMnaaBaaaleaacqWGbbqqaeqaaOGaeiikaGIaemyyaeMaeiykaKIaeyypa0tcfa4aaSaaaeaacqaIXaqmaeaacqWGIbGyaaWaaSaaaeaaiiGacqWFYoGydaahaaqabeaacqWFXoqyaaaabaGaeu4KdCKaeiikaGIae8xSdeMaeiykaKcaaOGaemyzau2aaWbaaSqabeaajuaGdaWcaaqaaiabdggaHbqaaiabdkgaIbaaliab=f7aHbaakiabdwgaLnaaCaaaleqabaGaeyOeI0Iae8NSdiMaemyzau2aaWbaaWqabKqbagaadaWcaaqaaiabdggaHbqaaiabdkgaIbaaaaaaaaGcbaaccaGae4NeI0IaeyOhIuQaeyipaWJaemyyaeMaeyipaWJaeyOhIukaaaaa@5428@

where Γ(α)=∫0∞tα−1e−tdt
 MathType@MTEF@5@5@+=feaafiart1ev1aqatCvAUfKttLearuWrP9MDH5MBPbIqV92AaeXatLxBI9gBaebbnrfifHhDYfgasaacPC6xNi=xH8viVGI8Gi=hEeeu0xXdbba9frFj0xb9qqpG0dXdb9aspeI8k8fiI+fsY=rqGqVepae9pg0db9vqaiVgFr0xfr=xfr=xc9adbaqaaeGacaGaaiaabeqaaeqabiWaaaGcbaGaeu4KdCKaeiikaGccciGae8xSdeMaeiykaKIaeyypa0Zaa8qmaeaacqWG0baDdaahaaWcbeqaaiab=f7aHjabgkHiTiabigdaXaaakiabdwgaLnaaCaaaleqabaGaeyOeI0IaemiDaqhaaOGaemizaqMaemiDaqhaleaacqaIWaamaeaacqGHEisPa0Gaey4kIipaaaa@41EB@ is the gamma function. Notice that X=eAb
 MathType@MTEF@5@5@+=feaafiart1ev1aqatCvAUfKttLearuWrP9MDH5MBPbIqV92AaeXatLxBI9gBaebbnrfifHhDYfgasaacPC6xNi=xH8viVGI8Gi=hEeeu0xXdbba9frFj0xb9qqpG0dXdb9aspeI8k8fiI+fsY=rqGqVepae9pg0db9vqaiVgFr0xfr=xfr=xc9adbaqaaeGacaGaaiaabeqaaeqabiWaaaGcbaGaemiwaGLaeyypa0Jaemyzau2aaWbaaSqabKqbagaadaWcaaqaaiabdgeabbqaaiabdkgaIbaaaaaaaa@3289@ is distributed according to a gamma distribution with shape parameter *α *and scale parameter 1/*β*. It can be demonstrated that a priori, the expected value of *A *is E(*A*) = *b*(*ψ *(*α*)-In *β*) where ψ(α)=Γ′(α)Γ(α)
 MathType@MTEF@5@5@+=feaafiart1ev1aqatCvAUfKttLearuWrP9MDH5MBPbIqV92AaeXatLxBI9gBaebbnrfifHhDYfgasaacPC6xNi=xH8viVGI8Gi=hEeeu0xXdbba9frFj0xb9qqpG0dXdb9aspeI8k8fiI+fsY=rqGqVepae9pg0db9vqaiVgFr0xfr=xfr=xc9adbaqaaeGacaGaaiaabeqaaeqabiWaaaGcbaacciGae8hYdKNaeiikaGIae8xSdeMaeiykaKIaeyypa0ZaaSaaaeaacuqHtoWrgaqbaiabcIcaOiab=f7aHjabcMcaPaqaaiabfo5ahjabcIcaOiab=f7aHjabcMcaPaaaaaa@3B7F@ is the digamma function and its variance is V(A)=b2(1α+12α2)
 MathType@MTEF@5@5@+=feaafiart1ev1aqatCvAUfKttLearuWrP9MDH5MBPbIqV92AaeXatLxBI9gBaebbnrfifHhDYfgasaacPC6xNi=xH8viVGI8Gi=hEeeu0xXdbba9frFj0xb9qqpG0dXdb9aspeI8k8fiI+fsY=rqGqVepae9pg0db9vqaiVgFr0xfr=xfr=xc9adbaqaaeGacaGaaiaabeqaaeqabiWaaaGcbaGaeeOvayLaeiikaGIaemyqaeKaeiykaKIaeyypa0JaemOyai2aaWbaaSqabeaacqaIYaGmaaGcdaqadaqaaKqbaoaalaaabaGaeGymaedabaacciGae8xSdegaaOGaey4kaSscfa4aaSaaaeaacqaIXaqmaeaacqaIYaGmcqWFXoqydaahaaqabeaacqaIYaGmaaaaaaGccaGLOaGaayzkaaaaaa@3E21@.

The conjugate posterior distribution for *A *is given by

fA(a|r1..rn)=g(r1..rn|a)f(a)∫−∞∞g(r1..rn|a)f(a)da−∞<a<∞
 MathType@MTEF@5@5@+=feaafiart1ev1aaatCvAUfKttLearuWrP9MDH5MBPbIqV92AaeXatLxBI9gBaebbnrfifHhDYfgasaacPC6xNi=xI8qiVKYPFjYdHaVhbbf9v8qqaqFr0xc9vqFj0dXdbba91qpepeI8k8fiI+fsY=rqGqVepae9pg0db9vqaiVgFr0xfr=xfr=xc9adbaqaaeGacaGaaiaabeqaaeqabiWaaaGcbaqbaeqabeGaaaqaaiabdAgaMnaaBaaaleaacqWGbbqqaeqaaOGaeiikaGIaemyyae2aaqqaaeaacqWGYbGCdaWgaaWcbaGaeGymaedabeaakiabc6caUiabc6caUiabdkhaYnaaBaaaleaacqWGUbGBaeqaaaGccaGLhWoacqGGPaqkcqGH9aqpjuaGdaWcaaqaaiabdEgaNjabcIcaOiabdkhaYnaaBaaabaGaeGymaedabeaacqGGUaGlcqGGUaGlcqWGYbGCdaWgaaqaaiabd6gaUbqabaWaaqqaaeaacqWGHbqyaiaawEa7aiabcMcaPiabdAgaMjabcIcaOiabdggaHjabcMcaPaqaamaapedabaGaem4zaCMaeiikaGIaemOCai3aaSbaaeaacqaIXaqmaeqaaiabc6caUiabc6caUiabdkhaYnaaBaaabaGaemOBa4gabeaadaabbaqaaiabdggaHbGaay5bSdGaeiykaKIaemOzayMaeiikaGIaemyyaeMaeiykaKIaemizaqMaemyyaegabaGaeyOeI0IaeyOhIukabaGaeyOhIukacqGHRiI8aaaaaOqaaGGaaiab=jHiTiabg6HiLkabgYda8iabdggaHjabgYda8iabg6HiLcaaaaa@6EED@

i.e.

fA(a|r1..rn)=enabe−Keabeabαe−βeab∫−∞∞enabe−Keabeabαe−βeabda−∞<a<∞
 MathType@MTEF@5@5@+=feaafiart1ev1aaatCvAUfKttLearuWrP9MDH5MBPbIqV92AaeXatLxBI9gBaebbnrfifHhDYfgasaacPC6xNi=xI8qiVKYPFjYdHaVhbbf9v8qqaqFr0xc9vqFj0dXdbba91qpepeI8k8fiI+fsY=rqGqVepae9pg0db9vqaiVgFr0xfr=xfr=xc9adbaqaaeGacaGaaiaabeqaaeqabiWaaaGcbaqbaeqabeGaaaqaaiabdAgaMnaaBaaaleaacqWGbbqqaeqaaOGaeiikaGIaemyyae2aaqqaaeaacqWGYbGCdaWgaaWcbaGaeGymaedabeaakiabc6caUiabc6caUiabdkhaYnaaBaaaleaacqWGUbGBaeqaaaGccaGLhWoacqGGPaqkcqGH9aqpjuaGdaWcaaqaaiabdwgaLnaaCaaabeqaamaalaaabaGaemOBa4MaemyyaegabaGaemOyaigaaaaacqWGLbqzdaahaaqabeaacqGHsislcqWGlbWscqWGLbqzdaahaaqabeaadaWcaaqaaiabdggaHbqaaiabdkgaIbaaaaaaaiabdwgaLnaaCaaabeqaamaalaaabaGaemyyaegabaGaemOyaigaaGGaciab=f7aHbaacqWGLbqzdaahaaqabeaacqGHsislcqWFYoGycqWGLbqzdaahaaqabeaadaWcaaqaaiabdggaHbqaaiabdkgaIbaaaaaaaaqaamaapedabaGaemyzau2aaWbaaeqabaWaaSaaaeaacqWGUbGBcqWGHbqyaeaacqWGIbGyaaaaaiabdwgaLnaaCaaabeqaaiabgkHiTiabdUealjabdwgaLnaaCaaabeqaamaalaaabaGaemyyaegabaGaemOyaigaaaaaaaGaemyzau2aaWbaaeqabaWaaSaaaeaacqWGHbqyaeaacqWGIbGyaaGae8xSdegaaiabdwgaLnaaCaaabeqaaiabgkHiTiab=j7aIjabdwgaLnaaCaaabeqaamaalaaabaGaemyyaegabaGaemOyaigaaaaaaaGaemizaqMaemyyaegabaGaeyOeI0IaeyOhIukabaGaeyOhIukacqGHRiI8aaaaaOqaaGGaaiab+jHiTiabg6HiLkabgYda8iabdggaHjabgYda8iabg6HiLcaaaaa@81F6@

The denominator integrates to bΓ(α+n)(β+k)α+n
 MathType@MTEF@5@5@+=feaafiart1ev1aqatCvAUfKttLearuWrP9MDH5MBPbIqV92AaeXatLxBI9gBaebbnrfifHhDYfgasaacPC6xNi=xH8viVGI8Gi=hEeeu0xXdbba9frFj0xb9qqpG0dXdb9aspeI8k8fiI+fsY=rqGqVepae9pg0db9vqaiVgFr0xfr=xfr=xc9adbaqaaeGacaGaaiaabeqaaeqabiWaaaGcbaGaemOyaiwcfa4aaSaaaeaacqqHtoWrcqGGOaakiiGacqWFXoqycqGHRaWkcqWGUbGBcqGGPaqkaeaadaqadaqaaiab=j7aIjabgUcaRiabdUgaRbGaayjkaiaawMcaamaaCaaabeqaaiab=f7aHjabgUcaRiabd6gaUbaaaaaaaa@3E2F@ and thus the posterior distribution of *A *is equal to

fA(a|r1..rn)=1b(β+k)α+nΓ(α+n)eab(α+n)e−(β+k)eab−∞<a<∞
 MathType@MTEF@5@5@+=feaafiart1ev1aqatCvAUfKttLearuWrP9MDH5MBPbIqV92AaeXatLxBI9gBaebbnrfifHhDYfgasaacPC6xNi=xI8qiVKYPFjYdHaVhbbf9v8qqaqFr0xc9vqFj0dXdbba91qpepeI8k8fiI+fsY=rqGqVepae9pg0db9vqaiVgFr0xfr=xfr=xc9adbaqaaeGacaGaaiaabeqaaeqabiWaaaGcbaqbaeqabeGaaaqaaiabdAgaMnaaBaaaleaacqWGbbqqaeqaaOGaeiikaGYaaqGaaeaacqWGHbqyaiaawIa7aiabdkhaYnaaBaaaleaacqaIXaqmaeqaaOGaeiOla4IaeiOla4IaemOCai3aaSbaaSqaaiabd6gaUbqabaGccqGGPaqkcqGH9aqpjuaGdaWcaaqaaiabigdaXaqaaiabdkgaIbaadaWcaaqaamaabmaabaacciGae8NSdiMaey4kaSIaem4AaSgacaGLOaGaayzkaaWaaWbaaeqabaGae8xSdeMaey4kaSIaemOBa4gaaaqaaiabfo5ahjabcIcaOiab=f7aHjabgUcaRiabd6gaUjabcMcaPaaakiabdwgaLnaaCaaaleqabaqcfa4aaSaaaeaacqWGHbqyaeaacqWGIbGyaaWccqGGOaakcqWFXoqycqGHRaWkcqWGUbGBcqGGPaqkaaGccqWGLbqzdaahaaWcbeqaaiabgkHiTmaabmaabaGae8NSdiMaey4kaSIaem4AaSgacaGLOaGaayzkaaGaemyzau2aaWbaaWqabKqbagaadaWcaaqaaiabdggaHbqaaiabdkgaIbaaaaaaaaGcbaaccaGae4NeI0IaeyOhIuQaeyipaWJaemyyaeMaeyipaWJaeyOhIukaaaaa@6D3C@

which belongs to the same family as the prior distribution with parameters *α' *= *α *+ *n *and *β' *= *β *+ *k*. *A posteriori*, the expected value of *A *is E(*A*) = *b*(*ψ *(*α *+ *n*) - In(*β *+ *k*) and its variance is V(A)=b2(1α+n+12(α+n)2)
 MathType@MTEF@5@5@+=feaafiart1ev1aqatCvAUfKttLearuWrP9MDH5MBPbIqV92AaeXatLxBI9gBaebbnrfifHhDYfgasaacPC6xNi=xH8viVGI8Gi=hEeeu0xXdbba9frFj0xb9qqpG0dXdb9aspeI8k8fiI+fsY=rqGqVepae9pg0db9vqaiVgFr0xfr=xfr=xc9adbaqaaeGacaGaaiaabeqaaeqabiWaaaGcbaGaeeOvayLaeiikaGIaemyqaeKaeiykaKIaeyypa0JaemOyai2aaWbaaSqabeaacqaIYaGmaaGcdaqadaqaaKqbaoaalaaabaGaeGymaedabaacciGae8xSdeMaey4kaSIaemOBa4gaaOGaey4kaSscfa4aaSaaaeaacqaIXaqmaeaacqaIYaGmcqGGOaakcqWFXoqycqGHRaWkcqWGUbGBcqGGPaqkdaahaaqabeaacqaIYaGmaaaaaaGccaGLOaGaayzkaaaaaa@4461@.
